# Thiamine Is Required for Virulence and Survival of *Pseudomonas syringae* pv. *tomato* DC3000 on Tomatoes

**DOI:** 10.3389/fmicb.2022.903258

**Published:** 2022-06-17

**Authors:** Jun Liu, Xuejiang Zhang, Siyi Deng, Hua Wang, Youfu Zhao

**Affiliations:** ^1^Institute of Plant Protection and Soil Fertilizer, Hubei Academy of Agricultural Sciences, Wuhan, China; ^2^Key Laboratory of Integrated Pest Management on Crops in Central China, Ministry of Agriculture, Wuhan, China; ^3^Hubei Key Laboratory of Crop Disease, Insect Pests and Weeds Control, Wuhan, China; ^4^Department of Plant Pathology, Irrigated Agriculture Research and Extension Center, Washington State University, Prosser, WA, United States

**Keywords:** *Pseudomonas syringae* pv. *tomato* DC3000, thiamine, cell growth, stress tolerance, virulence

## Abstract

*Pseudomonas syringae* pv. *tomato* DC3000 (*Pst*DC3000) is an important plant pathogen that infects tomatoes and *Arabidopsis*. Thiamine and its derivative thiamine pyrophosphate (TPP) are cofactors that play an important role in the growth and survival of many bacterial microorganisms. However, the role of thiamine-related genes has not been determined in *Pst*DC3000. Hence, to investigate the role of TPP in growth, resistance to stresses, and virulence of *Pst*DC3000, double and quadruple mutants of thiamine biosynthesis-related genes (*thiD/E, thiS/G*, and *thiD/E/S/G* deletion mutants) as well as a single mutant of a lipoprotein-related gene (*apbE*) were constructed. Our results showed that growth of the *thiD/E, thiS/G*, and *thiD/E/S/G* mutants in the mannitol-glutamate (MG) medium was significantly lower than that of the wild type (WT) and their growth could be restored to the WT level with the addition of exogenous thiamine, whereas mutation of the *apbE* gene did not affect its growth *in vitro*. While tolerance to acid, osmotic, and oxidative stresses for the double mutants was similar to the WT, tolerance to stresses for the *apbE* mutant was reduced as compared to the WT. In addition, all four mutants exhibited reduced virulence and growth in tomatoes. However, when the double and quadruple mutants were inoculated with exogenous thiamine, the virulence and growth rate of these mutants were restored to the WT level. These results indicated that the *thiD/E, thiS/G*, and *thiD/E/S/G* mutants exhibiting growth deficiency in *planta* are probably due to a lack of thiamine biosynthesis, thus reducing colonization in tomatoes. On the other hand, it is possible that the *apbE* mutant exhibited reduced stress tolerances, thus resulting in reduced colonization. Overall, our findings suggest that the thiamine biosynthetic (TBS) pathway plays an important role in the colonization and infection of *Pst*DC3000. Therefore, the thiamine biosynthetic pathway could be used as the target to develop new control measures for a bacterial spot in tomatoes.

## Introduction

*Pseudomonas syringae* pv. *tomato* DC3000 (*Pst*DC3000) is a model pathogen for studies in molecular mechanisms of bacterial pathogenesis and in plant–microbe interactions (Xin and He, 2013). *Pst*DC3000 causes bacterial speck disease on tomato and *Arabidopsis thaliana* by injecting effectors into plant cells through the type III secretion system (T3SS) and producing the phytotoxin coronatine (COR) (Zhao et al., 2003, 2006; Xie et al., 2019). Besides T3SS and phytotoxin coronatine (COR), extracellular protease, siderophore, and alginate all contribute to the virulence of *Pst*DC3000 (Brooks et al., 2004; Swingle et al., 2008; Vargas et al., 2013; Ishiga et al., 2018). Previous studies showed that guanosine tetra/pentaphosphate [(p)ppGpp] and RsmA affect virulence *via* regulation of T3SS, motility, siderophore, syringafactin, and alginate production in *Pst*DC3000 (Chatnaparat et al., 2015; Ge et al., 2019; Liu et al., 2020, 2021). In addition, mutations of (p)ppGpp-biosynthetic genes and *rsmA* significantly affected the expression of thiamine biosynthesis-related genes (Liu et al., 2020, 2021).

**Figure 1:**
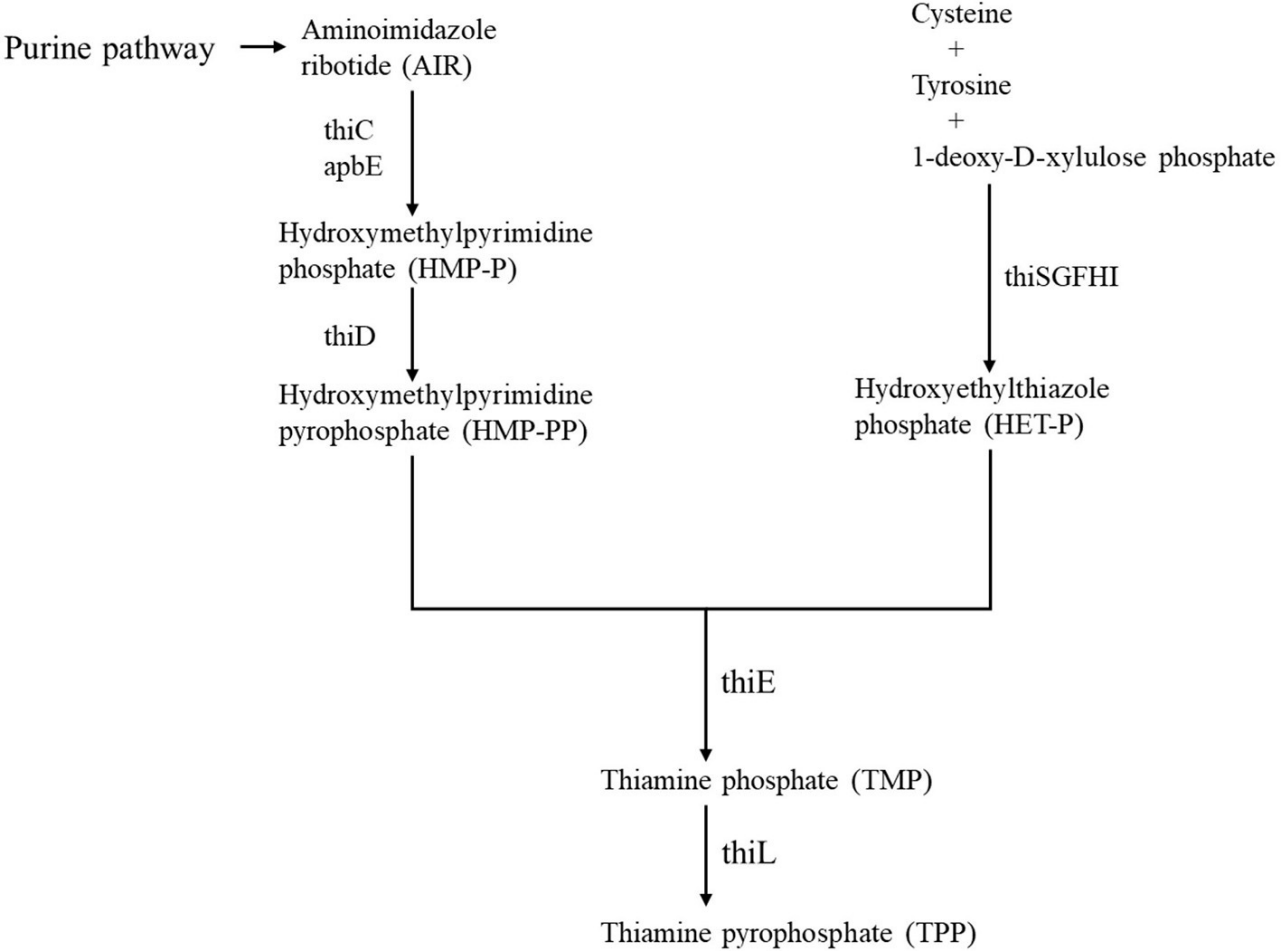
Diagram showing a putative thiamine biosynthesis pathway. Black arrows indicate thiamine biosynthetic (TBS) pathway. HMP-P, hydroxymethyl pyrimidine phosphate; HMP-PP, 4-amino-5-hydroxymethyl-2-methylpyrimidine diphosphate; HET-P, 5-(2-hydroxyethyl)-4-methylthiazole phosphate; TMP, thiamine monophosphate; AIR, aminoimidazole ribotide; TPP, thiamine pyrophosphate.

Vitamins of the B group are key precursors for the biosynthesis of essential enzyme cofactors that drive many metabolic processes in all the forms of life (Rodionov et al., 2017). The active form of vitamin B1 (thiamine), thiamine pyrophosphate (TPP), is an important cofactor for several anabolic and catabolic reactions in central metabolism (Begley and Ealick, 2010). Thiamine pyrophosphate (TPP)-dependent enzymes, including transketolase, pyruvate dehydrogenase, 2-oxoglutarate dehydrogenase, alpha-ketoglutarate dehydrogenase, and 1-deoxy-D-xylulose-5-phosphate synthase, catalyze essential cellular processes in bacteria, from central metabolism to biosynthesis of amino acids, cofactors, and lipids (Rapala-Kozik et al., 2012; Bunik et al., 2013; Osiezagha et al., 2013; Hwang et al., 2017; Chen et al., 2019). The thiamine biosynthetic (TBS) pathway is well-understood in bacteria, and several enzymes of the pathway have been identified (Rodionov et al., 2002; Jurgenson et al., 2009). Thiamine monophosphate (TMP) is formed by the coupling of two moieties, i.e., 4-amino-5-hydroxymethyl-2-methylpyrimidine pyrophosphate (HMP-PP) and 5-(2-hydroxyethyl)-4-methylthiazole phosphate (HET-P) by the thiamine-phosphate pyrophosphorylase (ThiE) (Tylicki et al., 2018). The aminoimidazole ribotide (AIR) derived from the purine pathway is catalyzed by hydroxymethyl pyrimidine synthase (ThiC) and lipoprotein (ApbE) to form hydroxymethyl pyrimidine phosphate (HMP-P), which is phosphorylated to form HMP-PP by the bifunctional HMP kinase/HMP-P kinase (ThiD) (Beck and Downs, 1998; Palmer and Downs, 2013; Rodionov et al., 2017). On the other hand, the thiazole moiety of thiamine HET-P is derived from tyrosine, cysteine, and 1-deoxy-D-xylulose phosphate and catalyzed by the products of the *thiF, thiS, thiG, thiH*, and *thiI* genes (Jurgenson et al., 2009; Rodionov et al., 2017). Lastly, TMP is phosphorylated by thiamine monophosphate kinase (ThiL) to form thiamine pyrophosphate (TPP) (Figure 1) (Begley et al., 1999; Melnick et al., 2004; Hwang et al., 2017). In addition to the TPP biosynthetic pathway, bacteria, such as *Escherichia coli* (*E. coli*), are able to salvage exogenous sources of thiamine to generate TPP by first converting thiamine to TMP thiamine kinase (Thik) and then subsequently generating TPP by adding an additional phosphate to TMP *via* ThiL (Rodionov et al., 2002; Melnick et al., 2004). Therefore, TPP is indispensable for bacterial survival, and enzymes involved in bacterial TPP biosynthesis might be important targets for disease control and prevention.

It has been previously reported that several enzymes of the TBS pathway are important for host infection. The protein encoded by *thiG* acts as an essential enzyme in the thiamine biosynthetic pathway, which plays a crucial role in tolerance to oxidative and heat stresses in *Fusarium oxysporum* (*F. oxysporum*) (Ruiz-Roldán et al., 2008) and contributes for full virulence in *Verticillium dahliae* and *Xanthomonas oryzae* (*X. oryzae*) pv. *oryzae* (Hoppenau et al., 2014; Yu et al., 2015). In *Listeria monocytogenes* (*L. monocytogenes*), mutation of the *thiD* gene significantly reduced bacterial replication in epithelial cells and inhibited infection (Schauer et al., 2009). In *Xanthomonas oryzae* pv. *oryzicola*, the *thiE* gene was reported to influence bacterial virulence (Zou et al., 2011). In addition, the *apbE* gene encoding a lipoprotein is involved in the conversion of AIR to the HMP moiety of TPP in *Salmonella typhimurium* (*S. typhimurium*) (Beck and Downs, 1998).

In this study, the open reading frames (ORFs) of ThiD (*thiD, pspto_4798*), ThiE (*thiE, pspto_4799*), ThiS (*thiS, pspto_0433*), ThiG (*thiG, pspto_0434*), and ApbE (*apbE, pspto_2105*) in the genome of *Pst*DC3000 (gene bank accession #: AE016853.1) were identified by performing BLAST with homologous enzymes from other organisms. In *Pst*DC3000, both the *thiDE* and *thiSG* genes are located adjacent to each other in the genome. However, the role of thiamine biosynthesis-related genes on the virulence of pathogens remains unknown. Thus, in order to determine whether thiamine biosynthesis-related genes and *apbE* gene affect the virulence of *Pst*DC3000, we constructed the *thiD/E, thiS/G, thiD/E/S/G*, and *apbE* mutants using splice overlap extension mutagenesis (Supplementary Figure S1) and their corresponding complementation strains in *Pst*DC3000 and assessed their growth *in vitro* and *in vivo*, stress tolerance, and virulence on tomato. Our findings would enhance our understanding of thiamine in the virulence of *Pst*DC3000.

## Materials and Methods

### Bacterial Strains and Culture Conditions

Bacterial strains and vectors used in this study are given in Supplementary Table S1. The WT *Pst*DC3000 and its mutants were routinely cultured on King's medium B (KB) or mannitol-glutamate (MG) medium (Park et al., 2010) with appropriate antibiotics at 28°C with shaking at 250 rpm. All the *E. coli* strains were grown in Luria-Bertani (LB) broth or agar medium with appropriate antibiotics at 37°C with shaking at 250 rpm. Bacterial growth was monitored by measuring the absorbance of cell suspensions at 600 nm. Antibiotics were supplied at the following final concentrations: 100 μg ml^−1^ rifampicin, 50 μg ml^−1^ kanamycin, 100 μg/ml ampicillin, 15 μg/ml tetracycline, and 100 μg/ml spectinomycin.

### Construction of Deletion Mutants

Deletion mutations of *thiD/E, thiS/G, thiD/E/S/G*, and *apbE* were generated using splice overlap extension mutagenesis (Supplementary Figure S1), as described previously (Ge et al., 2019). Briefly, approximately, 1 kb upstream and downstream fragments of *thiD/E, thiS/G*, and *apbE* in *Pst*DC3000 were amplified using primers unique to these regions (Supplementary Table S2). At the same time, to facilitate the screening of mutants, these primers also contained an extension sequence complementary to a kanamycin resistance cassette flanked with flippase (FLP) recombination target (FRT) sites from pKD13. The two PCR products contain ends overlapping with sequences at both ends of the kanamycin resistance cassette (Datsenko and Wanner, 2000). The FRT-Km-FRT forward and reverse primers were used amplified FRT-flanked kanamycin cassette. The two fragments were then joined with the kanamycin resistance cassette using overlap extension PCR amplified into a single fragment. The final fragment was cloned into the pTok2 suicide vector digested with SmaI, resulting in pTok2::Δ*thiD/E*, pTok2::Δ*thiS/G*, and pTok2::Δ*apbE*, respectively. Each of these plasmids was electroporated into *Pst*DC3000. Subsequently, transformants were plated on KB plates containing rifampicin and kanamycin. Those kanamycin-resistant, tetracycline-sensitive mutants were selected. Gene disruption was confirmed using PCR, with primers specific to the sequences flanking each of the genes. To generate markerless mutants, plasmid pFLP2-omega expressing FLP recombinase was electroporated into the mutant strains. Transformants were plated on KB plates containing spectinomycin to allow FLP recombinase-mediated recombination between the FRT sites, resulting in the loss of the kanamycin resistance cassette. The pFLP2-omega plasmid was cured of the mutants by plating on KB plates containing 10% sucrose. Markerless deletion strains were confirmed by PCR. In order to generate a *thiD/E/S/G* quadruple deletion mutant, plasmid pTok2::Δ*thiD/E* was transferred to the *thiS/G* markerless mutant strain *via* conjugation.

### Complementation of Mutants

For complementation of the *thiD/E, thiS/G*, and *apbE* mutants, about 2 kb fragment containing the native promoter and the *thiD/E, thiS/G*, and *apbE* genes was amplified by PCR and cloned into the pUCP18 vector to yield plasmids pThiD/E, pThiS/G, and pApbE, respectively. These plasmids were sequenced at the University of Illinois at Urbana-Champaign core sequencing facility. The final plasmids were introduced into the corresponding deletion mutants and *Pst*DC3000 by electroporation. Transformants were selected on KB agar plates amended with 100 μg/ml ampicillin. Finally, the complementation of mutants was confirmed by PCR and used for further study. All the primer sequences used in this study are given in Supplementary Table S2 (see Supplementary Materials).

### Growth Curve Assay

*Pseudomonas syringae* pv. *tomato* DC3000 WT strain, mutants, and corresponding complementation strains were cultured in KB broth at 28°C for 18 h with shaking at 250 rpm. All the strains were harvested and resuspended to OD_600_ = 0.01 in fresh KB. Bacterial strains were grown at 28°C and aliquots of the culture were taken after 2, 4, 6, 8, 10, and 24 h. Furthermore, all the strains were harvested and resuspended to OD_600_ = 0.01 in MG medium with 100 μg/ml thiamine (Fisher Scientific, Waltham, MA, USA) or without thiamine at 28°C and aliquots of the culture were taken after 24, 48, and 72 h. These experiments were performed in triplicate and repeated three times.

### Stress Tolerance (Acid, Oxidative, and Osmotic) Assays

Growth assays were performed using a previously described procedure to detect stress response (Ge et al., 2018). Briefly, overnight bacterial cells were harvested by centrifugation and washed twice using phosphate-buffered saline (PBS). After the final wash, the pellet was resuspended in PBS and adjusted to a concentration of OD_600_ = 0.1. Bacterial cells of overnight cultures in KB were washed and resuspended to OD_600_ = 0.01 in KB liquid medium with pH 5.5, 0.5 M NaCl, and 0.5 mM hydrogen peroxide (H_2_O_2_), respectively. Cells were incubated at 28°C for 24 h and bacterial growth was monitored by measuring OD_600_. Meanwhile, bacterial cells cultured in a normal KB medium were used for normalization. These experiments were performed in triplicate and repeated three times.

### Spot Dilution Plate Assay

Overnight bacterial cultures were harvested by centrifugation and cells were washed two times using PBS. Bacterial cells were resuspended in PBS and were adjusted to an initial concentration of OD_600_ = 0.1 for assays on KB plates. Ten-fold serial dilution of the bacterial suspension was made using PBS. For each dilution, 5 μl was spotted on the KB plates with or without acid, oxidative, and osmotic stresses, and was incubated at 28°C for 48 h. Growth on normal KB plates was used as a control to show similar growth of the WT and the mutants, whereas the growth of mutants was compared with that of the WT on KB plates with acid, oxidative, and osmotic stresses. If the growth of the mutant was observed at least one dilution lower or higher than that of the WT, this indicated that the mutant was more resistant or sensitive to acid, oxidative, and osmotic stresses and vice versa. The experiments were performed in triplicate and were repeated at least three times.

### Virulence Assay and Bacterial Growth in Tomato

Virulence of *Pst*DC3000 and its mutants on tomatoes was evaluated using an injection of tomato leaves, as described previously (Ge et al., 2019). For injection of tomato leaves, overnight cultures of *Pst*DC3000, mutants, and complementation strains were harvested by centrifugation and adjusted to a concentration of about 5 × 10^4^ colony-forming unit (CFU)/ml [diluted from original suspension at OD_600_ = 0.1 (~10^8^ CFU per milliliter)] with PBS. Each strain was inoculated into 10 tomato leaves (Tomato, *Solanum lycopersicum* “Big Daddy Hybrid” plants were grown in a greenhouse) using a needleless syringe, which were then observed for disease symptoms at 7 days postinoculation (dpi). For bacterial growth on tomato leaves, bacteria were recovered from plants by taking three samples from three leaves at the site of infiltration using a disk punch (three disks per strain) at 0, 1, 3, and 5 dpi. Leaf disks were homogenized by mechanical disruption using pestles in PBS. Serial 10-time dilutions of the homogenates were plated on KB plates containing rifampicin antibiotic and the number of CFUs per disk (cm^2^) was calculated. The experiment was repeated three times.

### Statistical Analyses

SPSS statistics version 26 was used to perform a one-way ANOVA and least significant difference (LSD) test to determine the significant difference in the growth curve, stress tolerance (acid, oxidative, and osmotic) assays, and bacterial colonization. Differences with *P* < 0.05 were considered statistically significant.

## Results

### Effect of Thiamine on Bacterial Growth *in vitro*

**Figure 2:**
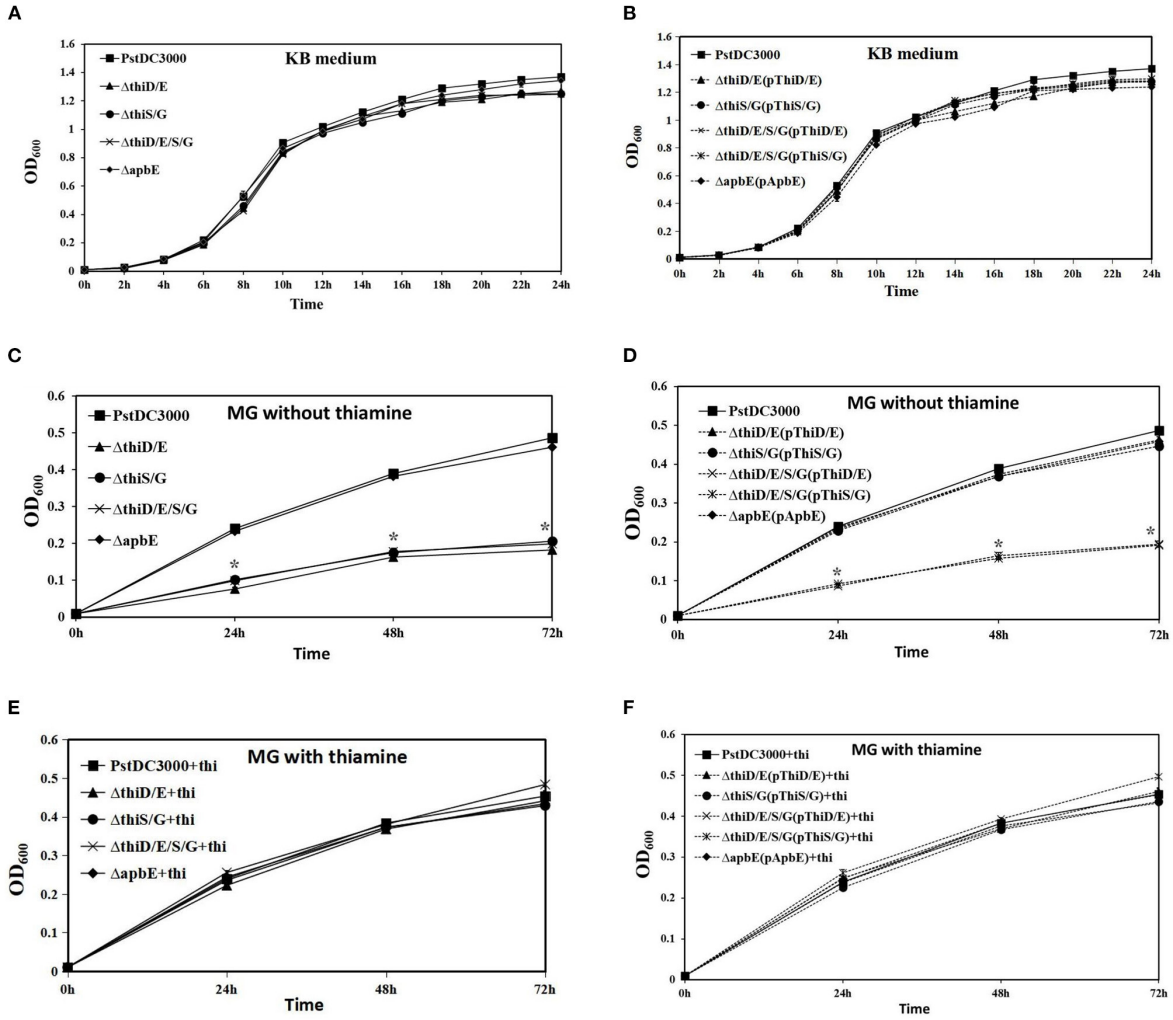
Thiamine-related genes are required for the growth of *Pseudomonas syringae* pv. *tomato* DC3000 (*Pst*DC3000) in minimum medium. **(A)** Growth of *Pst*DC3000 wild type (WT), the *thiD/E, thiS/G, thiD/E/S/G*, and *apbE* mutant strains in King's B medium (KB). **(B)** Growth of complementation strains in KB. **(C)** Growth of all the mutant strains in mannitol-glutamate (MG) medium. **(D)** Growth of complementation strains in MG medium. **(E)** Growth of all the mutant strains in MG medium with exogenous 100 μg/ml thiamine. **(F)** Growth of complementation strains in MG medium with exogenous 100 μg/ml thiamine. The solid line represents mutant strains; the dashed line represents complementation strains; and the vertical bars represent SDs. “+thi:” added exogenous 100 μg/ml thiamine. Experiments were performed in triplicate and repeated three times. Statistical tests were performed at 24, 48, and 72 h. Statistically significant differences were determined by one-way ANOVA and *P*-values were < 0.05 [an asterisk (*) indicates *P* < 0.05, mutant and complementation strains vs. the WT *Pst*DC3000].

Thiamine (vitamin B1) is an essential cofactor for carbohydrate and branched-chain amino acid metabolic enzymes in all the organisms (Settembre et al., 2003; Du et al., 2011). To investigate whether *thiD/E, thiS/G*, and *apbE* had an effect on *in vitro* growth, we examined the growth of the *thiD/E, thiS/G, thiD/E/S/G*, and *apbE* deletion mutants and their complementation strains in the rich medium (KB) and limited nutrition medium (MG). In KB, the growth of the *thiD/E, thiS/G*, and *thiD/E/S/G* deletion mutants and their complementation strains were similar to that of *Pst*DC3000 (Figures 2A,B). However, growth of the *thiD/E, thiS/G*, and *thiD/E/S/G* mutants was significantly (*P* < 0.05) reduced as compared with *Pst*DC3000 in MG medium without exogenous thiamine (Figure 2C). All the complementation strains, except the *thiD/E/S/G* mutant complementation (*P* < 0.05) with either the *thiD/E* or *thiS/G* genes, restored growth levels similar to these of the *Pst*DC3000 in MG medium without exogenous thiamine (Figure 2D). When supplemented with exogenous thiamine in MG medium, wild type, *thiD/E, thiS/G*, and *thiD/E/S/G* mutants and its complementation strains exhibited similar growth as *Pst*DC3000 (Figures 2E,F), suggesting that growth arrest of the mutant strains was caused by thiamine starvation. In contrast, the growth of the *apbE* deletion mutant and its complementation strain was similar to *Pst*DC3000 in both the KB and MG media (Figure 2). These results suggest that the *thiD/E* and *thiS/G* genes are required for the growth of *Pst*DC3000 in an MG medium.

### Role of Thiamine in Acid, Osmotic, and Oxidative Tolerances

**Figure 3:**
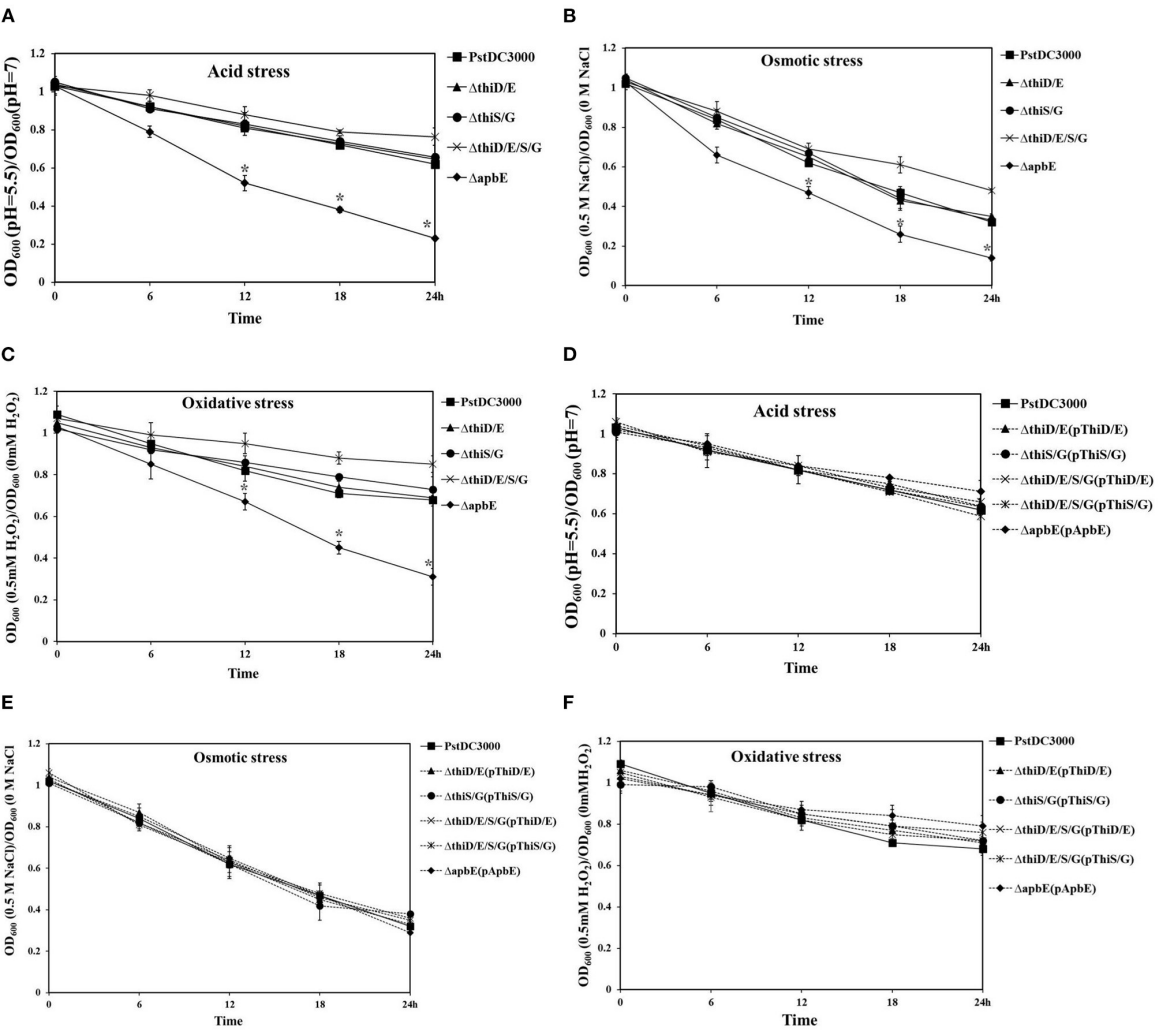
Thiamine-related genes affected tolerance to acid, oxidative, and osmotic stresses of *Pst*DC3000. **(A)** *Pst*DC3000 wild type, the *thiD/E, thiS/G, thiD/E/S/G*, and *apbE* mutant cells of overnight cultures in KB were washed and resuspended to OD_600_ = 0.01 in KB liquid medium with pH 5.5. The phenotype was observed after incubation at 28°C for 24 h and bacterial growth was monitored by measuring OD_600_. **(B)** All the mutant cells were grown in KB liquid medium with 0.5 M NaCl and incubated at 28°C for 24 h. **(C)** All the mutant cells were grown in KB liquid medium with 0.5 mM hydrogen peroxide (H_2_O_2_) and incubated at 28°C for 24 h. **(D)** All the complementation strains were grown in KB liquid medium with pH 5.5. **(E)** All the complementation strains were grown in KB liquid medium with 0.5 M NaCl. **(F)** All the complementation strains were grown in KB liquid medium with 0.5 mM H_2_O_2._ The solid line represents mutant strains; the dashed line represents complementation strains; and the vertical bars represent SDs. Experiments were performed in triplicate and repeated three times. Statistical tests were performed and statistically significant differences were determined by one-way ANOVA and *P*-values were < 0.05 [an asterisk (*) indicates *P* < 0.05, mutant and complementation strains vs. the WT *Pst*DC3000].

**Figure 4:**
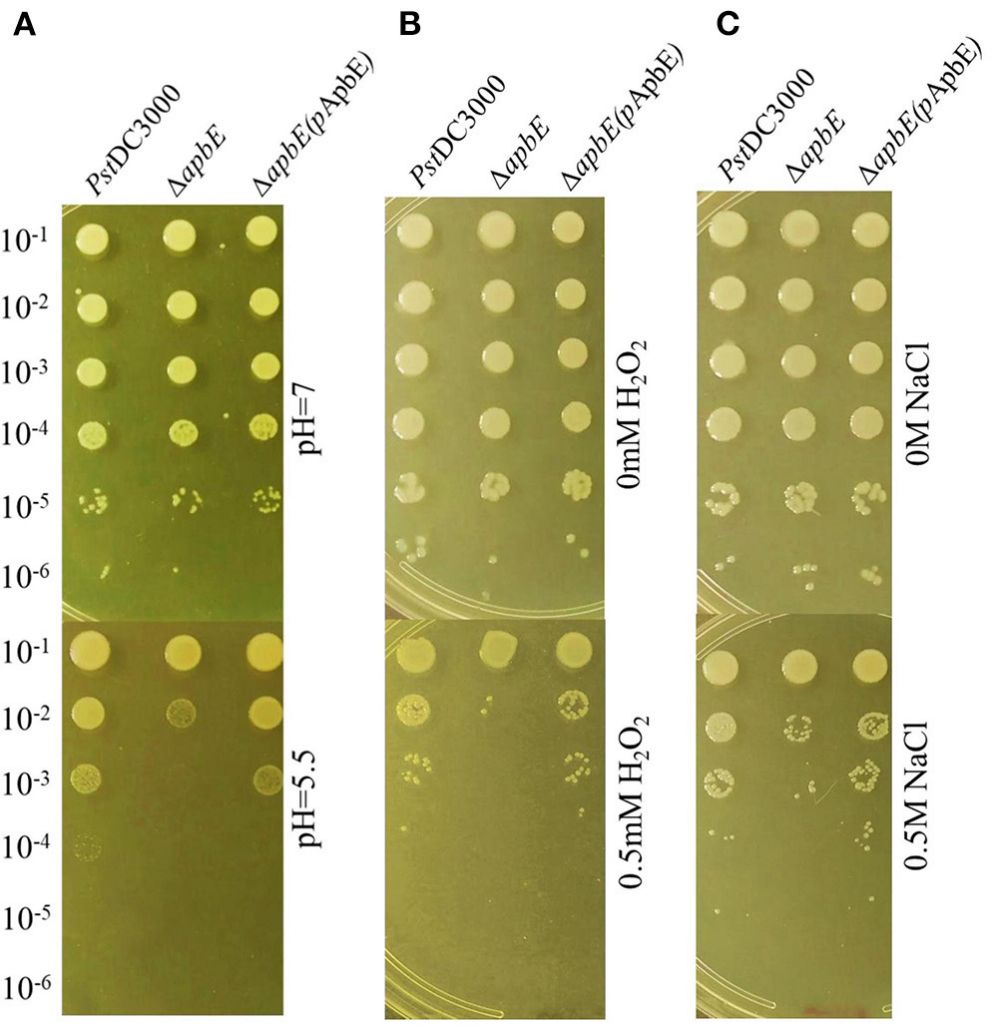
ApbE plays an important role in stress responses in *Pst*DC3000. **(A)** Acid stress. **(B)** Oxidative stress. **(C)** Osmotic stress. Serial 10-fold dilutions were made from optical density at 600 nm (OD_600_) = 0.1 and 5 μl for each dilution was added to plates containing acid, oxidative, and osmotic stresses, respectively. Photographs were taken 48 h postincubation. Experiments were performed in triplicate and repeated three times.

To test whether thiamine plays an important role in stress tolerance, including acid, osmotic, and oxidative responses, we performed growth assays in KB liquid medium. Growth of the *thiD/E* and *thiS/G* mutants was essentially unaffected after being incubated in KB liquid medium at pH 5.5, with 0.5 M NaCl or 0.5 mM H_2_O_2_, and growth of the *thiD/E/S/G* mutant showed slightly increase, whereas growth of the *apbE* mutant significantly (*P* < 0.05) reduced as compared with that of *Pst*DC3000 (Figures 3A–C). The growth of all the complementation strains was similar with *Pst*DC3000 in KB liquid medium at pH 5.5, with 0.5 M NaCl or 0.5 mM H_2_O_2_ (Figures 3D–F). Spot dilution assays on KB plates further illustrated the role of *apbE* in acid, osmotic, and oxidative tolerances. On KB plates at pH = 7, with no NaCl or H_2_O_2_, respectively, growth of the *apbE* mutant was equivalent to those of the WT strain (Figure 4). When plated on KB at pH = 5.5, with 0.5 M NaCl or 0.5 mM H_2_O_2_, the growth of the *apbE* mutant was reduced at least 10-fold as compared with the WT (Figure 4). These results indicate that the *apbE* gene plays a role in stress responses in *Pst*DC3000.

### Role of Thiamine in *Pst*DC3000 Virulence

**Figure 5:**
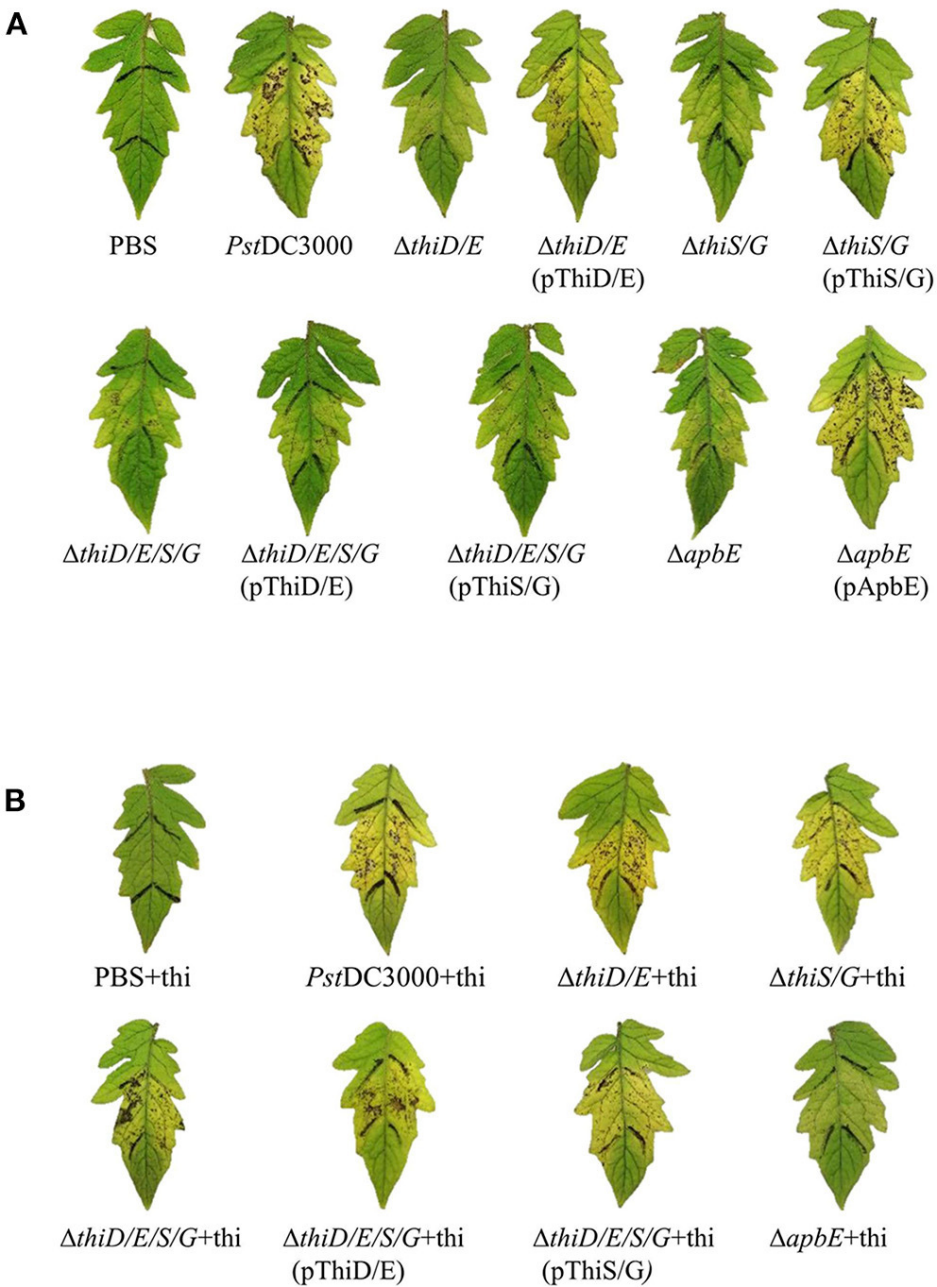
Thiamine-related genes contribute to *Pst*DC3000 virulence. **(A)** Tomato leaves inoculated by infiltration with *Pst*DC3000 wild type and the *thiD/E, thiS/G, thiD/E/S/G, apbE* mutants, and complementation strains. Phosphate-buffered saline (PBS) was used as a negative control. **(B)** *Pst*DC3000 wild type, the *thiD/E, thiS/G, thiD/E/S/G* mutants, and their corresponding complementation strains with exogenous 100 μg/ml thiamine inoculated into tomato leaves. Pictures were taken at 7 days postinoculation (dpi). “+thi:” added exogenous 100 μg/ml thiamine. Each strain was inoculated onto 10 tomato leaves with a needleless syringe in each experiment. Experiments were repeated three times.

To determine the effect of thiamine in virulence of *Pst*DC3000 on tomatoes, *Pst*DC3000 wild type, the *thiD/E, thiS/G, thiD/E/S/G*, and *apbE* mutants were infiltrated into tomato leaves at a concentration of 5 × 10^4^ CFU/ml. The *Pst*DC3000 WT strain exhibited typical necrotic spots with chlorosis on leaves of tomato plants at 7 dpi, whereas the *thiD/E, thiS/G, thiD/E/S/G*, and *apbE* mutant strains all exhibited reduced virulence (Figure 5A). All the complementation strains, except for the *thiD/E/S/G* mutant complementation with either the *thiD/E* or *thiS/G* genes, restored virulence similar to these of the *Pst*DC3000 (Figure 5A). On the other hand, to determine whether thiamine could restore the ability of the mutants to cause disease, we inoculated plants with the mutants and complementation strains by supplementing exogenous thiamine at 100 μg/ml. As shown in Figure 5B, these mutants and complementation strains, except the *apbE* mutant, all exhibited similar virulence as that of *Pst*DC3000 (Figure 5B). These results indicate that thiamine is required for full virulence of *Pst*DC3000.

### Deletion of the *thiD/E, thiS/G*, and *apbE* Genes in *Pst*DC3000 Reduced Bacterial Growth on Tomato Leaves

**Figure 6:**
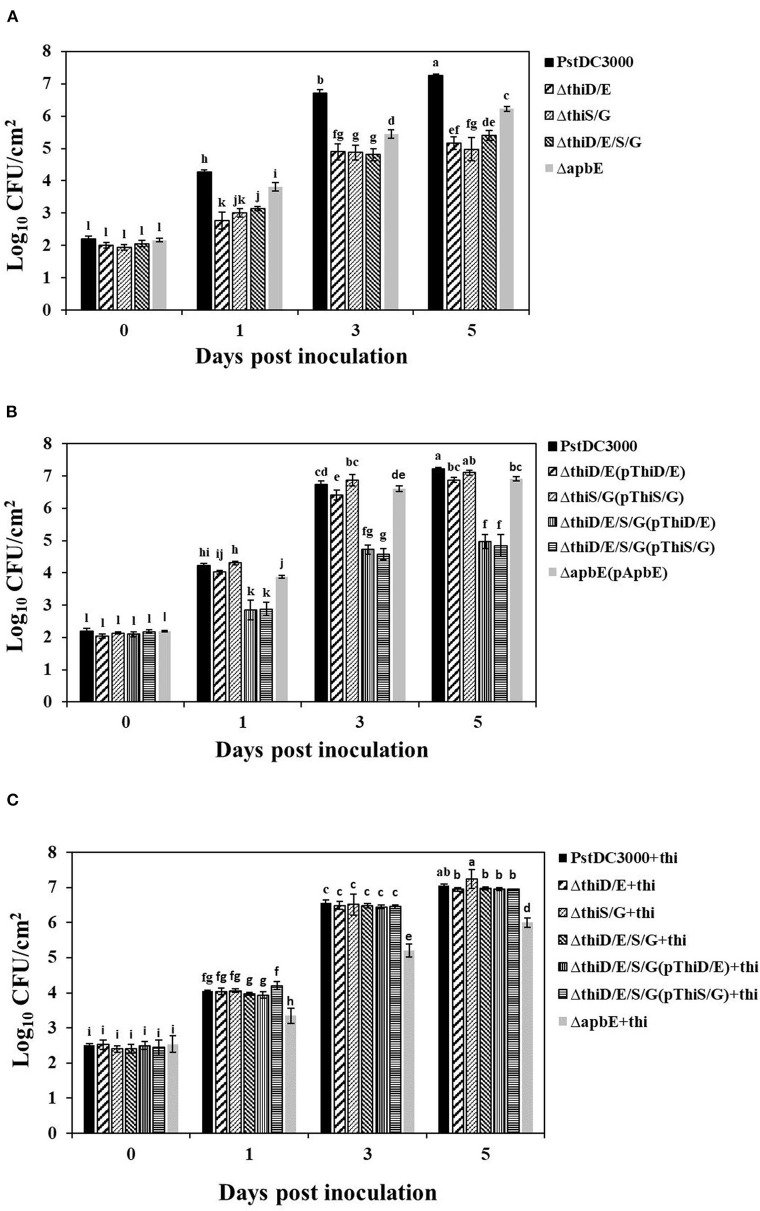
Thiamine is required for bacterial growth of *Pst*DC3000 in tomato leaves. **(A)** *Pst*DC3000 wild type and the *thiD/E, thiS/G, thiD/E/S/G*, and *apbE* mutant strains. **(B)** *Pst*DC3000 wild type and all the complementation strains. **(C)** *Pst*DC3000 wild type and the *thiD/E, thiS/G, thiD/E/S/G*, and *apbE* mutants with exogenous 100 μg/ml thiamine. Bacterial growth was monitored at 0, 1, 3, and 5 dpi. “+thi:” added exogenous 100 μg/ml thiamine. Each strain was inoculated onto 10 tomato leaves with a needleless syringe in each experiment. Experiments were repeated three times. Data represent the means of three replicates ± SDs (error bars). Different lowercase letters indicate a significant difference between the strains. Statistically significant differences were determined by one-way ANOVA and *P*-values were < 0.05.

To determine whether reduced virulence by the mutants was correlated with bacterial growth on tomato leaves, bacterial growth was monitored *in planta* at 0, 1, 3, and 5 dpi. Bacterial growth of the *thiD/E, thiS/G, thiD/E/S/G*, and *apbE* mutants was about 100-fold lower than (*P* < 0.05) those of the *Pst*DC3000 at 3 and 5 dpi (Figure 6A), whereas growth of the *apbE* mutant was about 10-fold lower than (*P* < 0.05) those of the *Pst*DC3000 at 3 and 5 dpi (Figure 6A). The reduced bacterial growth of the mutants could be completely rescued in the complementation strains, except for the *thiD/E/S/G* mutant complementation with either the *thiD/E* or *thiS/G* genes (*P* < 0.05) (Figure 6B). Similarly, when the double mutants and the *thiD/E/S/G* mutant complementation, with either the *thiD/E* or *thiS/G* genes, were infiltrated with supplemented exogenous thiamine, the growth of these strains was similar to *Pst*DC3000, whereas the growth of the *apbE* mutant was lower than (*P* < 0.05) those of the *Pst*DC3000 (Figure 6C). These results suggested that thiamine starvation may lead to reduced growth of the *thiD/E, thiS/G*, and *thiD/E/S/G* mutants in plants, whereas reduced ability for stress tolerance in the *apbE* mutant might be the cause of its reduced growth in tomatoes.

## Discussion

The TBS pathway is essential for the proper function of animals, plants, and microorganisms, as it plays a crucial role in cellular progress, including carbohydrate, lipid, amino acid, and nucleic acid metabolisms (Hwang et al., 2017; Chen et al., 2019). The TBS pathway in *E. coli* (Rodionov et al., 2002; Melnick et al., 2004) and *Salmonella typhimurium* (Webb et al., 1998) has been extensively studied through biochemical analysis and comparative genomes. In previous studies, 12 genes have been identified for thiamine biosynthesis in prokaryotes. These include *thiF, thiS, thiG, thiH, thiI*, and *dxs*, which are required for thiazole biosynthesis, *thiC*, which is involved in pyrimidine biosynthesis, *thiE*, which is required for linking of thiazole and pyrimidine, and *thiD, thiM, thiL*, and *thiK*, which are kinase genes (Jurgenson et al., 2009; Hwang et al., 2017). However, there are no reports about the role of thiamine biosynthesis during the interaction between bacteria and host plants in *Pst*DC3000. In present study, our results showed that growth of the *thiD/E, thiS/G*, and *thiD/E/S/G* mutants in MG medium was significantly decreased in MG medium, whereas mutation of the *apbE* gene did not affect its growth *in vitro*, while tolerance to acid, osmotic, and oxidative stresses for the *apbE* mutant was reduced as compared to the WT. In addition, all the mutants exhibited reduced virulence and growth in tomatoes. Therefore, our results suggest that the thiamine biosynthetic (TBS) pathway plays an important role in the colonization and infection of *Pst*DC3000.

Thiamine (vitamin B1) is an essential cofactor for carbohydrate metabolism (Hwang et al., 2017; Chen et al., 2019) and biosynthesis and degradation of the branched-chain amino acids, including isoleucine, leucine, and valine (Schauer et al., 2009). Therefore, the availability of thiamine has a key effect on the growth of many organisms, e.g., *X. oryzae* pv. *oryzae* (Yu et al., 2015), *Listeria monocytogenes* (Madeo et al., 2012), and *F. oxysporum* (Ruiz-Roldán et al., 2008). Consistent with other organisms, *thiD/E, thiS/G*, and *thiD/E/S/G* play an important role in the growth of a low nutrition environment, suggesting that thiamine biosynthesis is required for maintaining the growth of *Pst*DC3000. On the other hand, the growth of the *apbE* mutant was not affected, which is inconsistent with an earlier report on *S. typhimurium* (Beck and Downs, 1998). This result indicates that the *abpE* gene may not be directly involved in the biosynthesis of thiamine in *Pst*DC3000.

The stress response of microorganisms is indispensable since the living environment of microorganisms is constantly changing (Vorob'eva, 2003; Jordan et al., 2008; Swiecilo and Zych-Wezyk, 2013). Therefore, microorganisms must continuously sense and respond to environmental stimuli, e.g., osmotic, oxidative, and acid stresses (Moat et al., 2003; Bremer and Krämer, 2019). Many studies have shown that stress resistance mechanisms play an important role in virulence. As an example, OxyR of *Pst*DC3000 plays an important role in virulence by regulating the expression of genes related to oxidative stress (Ishiga and Ichinose, 2016). The DnaK/DnaJ of *S. typhimurium* is essential for stress resistance and invasion of epithelial cells (Takaya et al., 2004). Moreover, the *thiT* deletion mutant is necessary for acid tolerance in *L. monocytogenes* and thiamine starved cells are more acid-sensitive than the wild type (Madeo et al., 2012). In contrast, the *thiD/E* and *thiS/G* double mutants exhibited similar stress tolerance as that of *Pst*DC3000, indicating that thiamine is not a key factor in stress tolerance in *Pst*DC3000. However, the *thiD/E/S/G* quadruple mutant showed increased acid, osmotic, and oxidative tolerances, suggesting either a synergistic effect between them or some unknown functions of the intermediates in the pathways they are involved in. On the other hand, the *apbE* mutant exhibited significantly reduced acid, osmotic, and oxidative tolerances as compared to *Pst*DC3000, indicating that ApbE might play an important role in protecting the bacteria from stress responses.

It has been reported that thiamine-related genes play a critical role in virulence. The *thiD* mutation of *L. monocytogenes* exhibited significantly reduced bacterial replication in epithelial cells and infection (Schauer et al., 2009). In *X. oryzae* pv. *oryzicola*, the *thiE* gene was reported to influence bacterial virulence (Zou et al., 2011). The protein encoded by *thiG* acts as an essential enzyme in the thiamine biosynthetic pathway, which plays a crucial role in the virulence of *Verticillium dahliae* (Hoppenau et al., 2014). In *X. oryzae* pv. *oryzae*, the *thiG* mutant showed reduced virulence, while bacterial growth *in planta* was similar to the WT (Yu et al., 2015). In this study, the *thiD/E, thiS/G*, and *thiD/E/S/G* mutants exhibited significantly reduced virulence and bacterial growth *in planta*, which could be restored by supplementing with thiamine in an extracellular environment. These results demonstrated that lack of thiamine biosynthesis leads to growth deficiency, thus disease symptoms *in planta*. In contrast, the *apbE* mutant was normal growth *in vitro*, but still exhibited reduced virulence and bacterial growth *in planta*, suggesting that the loss of virulence of the *apbE* mutant may be due to reduced stress tolerance in tomatoes.

In summary, we provide evidence that the TBS pathway and ApbE play an important role in growth, colonization, and disease development in *Pst*DC3000. These results provide new insight into the role of the thiamine-related genes in *Pst*DC3000 virulence. In the future, we will investigate whether stress tolerance-related genes are directly or indirectly regulated by ApbE. Taken together, the results of this study demonstrate that thiamine plays an important role in the infection and survival of *Pst*DC3000 and provides a new target for the development of new drugs to prevent and control tomato bacterial spots.

## Data Availability Statement

The original contributions presented in the study are included in the article/Supplementary Materials, further inquiries can be directed to the corresponding authors.

## Author Contributions

JL, YZ, and HW designed the study. JL, XZ, and SD performed the study and analyzed the data. JL wrote the manuscript. YZ and HW revised the manuscript. All authors have read and approved the final version of the manuscript.

## Funding

This study was financially supported by the National Key Research and Development Program of China (2019YFD1002000), the Key Technology Research and Demonstration Project of the Hubei Agricultural Science and Technology Innovation Center (2020-620-000-002-07) and Postdoctoral Innovative Research Positions in Hubei Province.

## Conflict of Interest

The authors declare that the research was conducted in the absence of any commercial or financial relationships that could be construed as a potential conflict of interest.

## Publisher's Note

All claims expressed in this article are solely those of the authors and do not necessarily represent those of their affiliated organizations, or those of the publisher, the editors and the reviewers. Any product that may be evaluated in this article, or claim that may be made by its manufacturer, is not guaranteed or endorsed by the publisher.
