# Supplementary material for: Thiamine Is Required for Virulence and Survival of Pseudomonas syringae pv. tomato DC3000 on Tomatoes
Source: Front Microbiol. 2022 Jun 17;13:903258. doi: 10.3389/fmicb.2022.903258 (PMC9247456; doi:10.3389/fmicb.2022.903258)
Supplement: Supplementary file 1 [file Data_Sheet_1.PDF]

Supplementary Material

Table S1 Bacterial strains and plasmids used in this study.

Strains, plasmid	Description	Reference or source
Pseudomonas syringae* pv. *tomato		
DC3000	Wild type, Rif^r spontaneous resistance	
Δ*thiD/thiE*	Km^r, thiD and thiE::Kan, *Pst*DC3000 derivative	This study
Δ*thiS/thiG*	Km^r, thiS and thiG::Kan, *Pst*DC3000 derivative	This study
Δ*thiD/thiE/thiS/thiG*	Km^r, thiD, thiE, thiS and thiG::Kan, *Pst*DC3000 derivative	This study
Δ*apbE*	Km^r, apbE::Kan, DC3000 derivative	This study
Escherichia coli		This study
DH10B	*F-mcrA Δ(mrr-hsdRMS-mcrBC) Φ80lacZ ΔM15 ΔlacX74 recA1 endA1 araΔ139 Δ(ara, leu)7697 galU galK λ-rpsL (StrR)nupG*	Invitrogen (Carlsbad, CA, USA)
**Plasmids**		
pUCP18	*E.coli-pseudomonas* shuttle vector, Ap^r	Norrander et al., 1983
pTok2	ColE1 replicon, suicide plasmid, Tc^r	Kitten and Willis, 1996
pKD13	FRT-Kan-FRT, oriR6K, Ap^r, Km^r	Datsenko and Wanner, 2000
pFLP2-omega	Suicide vector encoding flp recombinase, *sacB* , Sp^r	Chatnaparat et al., 2015
pTok2::Δ*thiD/thiE*	Δ*thiD*/*E*::Kan from overlapping PCR cloned into pTok2, Tc^r, Km^r	This study
pTok2::Δ*thiS/thiG*	Δ*thiS/G*::Kan from overlapping PCR cloned into pTok2, Tc^r, Km^r	This study
pTok2::Δapb*E*	Δ*apbE*::Kan from overlapping PCR cloned into pTok2, Tc^r, Km^r	This study
pThiD/E	2018-bp fragment containing *thiD* and *thiE* gene with native promoter cloned into pUCP18, Ap^r	This study
pThiS/G	2024-bp fragment containing *thiS* and *thiG* gene with native promoter cloned into pUCP18, Ap^r	This study
pApbE	1804-bp fragment containing *apbE* gene with native promoter cloned into pUCP18, Ap^r	This study

Rif^r, Km^r, Tc^r, Ap^r and Sp^r indicate rifampicin, kanamycin, tetracycline, ampicillin and spectinomycin resistance, respectively.

Table S2. Primers used in this study

Primer	Sequences (5′–3′)
**Mutagenesis**	
thiD/E F1	AACCTTCGCTGCTGATACGC
thiD/E R1	GAAGCAGCTCCAGCCTACACAGTAATGGCGGGAGCGGCAT
thiD/E F2	GGTCGACGGATCCCCGGAATGCCGAAACCCCACAGGAAGT
thiD/E R2	CAGGGTATGTTTGGCGAAGG
thiS/G F1	GTGCTCGGCAAACTTCTGGT
thiS/G R1	GAAGCAGCTCCAGCCTACACATTCAAAGGATTCACCGTTC
thiS/G F2	GGTCGACGGATCCCCGGAATACGAAAACTCTATGCCAGCG
thiS/G R2	ATCAAAGCCAATGCCGAAGC
apbE F1	CAGATAGACCGAAACACCAC
apbE R1	GAAGCAGCTCCAGCCTACACCAATCCTGTAAAACCCTCGC
apbE F2	GGTCGACGGATCCCCGGAATCTTTATCATTCGCAGCAACG
apbE R2	CTCGTATTCCTCGTTGTGAC
FRT-Km-FRT F	GTGTAGGCTGGAGCTGCTTC
FRT-Km-FRT R	ATTCCGGGGATCCGTCGACC
**Complementation**	
thiD/E-compF (EcoRI)	AGTCGAATTCTCAACAGATAGCGCATTGAA
thiD/E-compR (BamHI)	AGTCGGATCCTGCTTTATTGAGTGGCATGA
thiS/G-compF (EcoRI)	AGTCGAATTCGAACCAGGATTCGACCTTG
thiS/G-compR (BamHI)	AGTCGGATCCATTTCCAGCAACGAATGG
apbE-compF (EcoRI)	AGTCGAATTCGATGTAGGCACCCGAGAATA
apbE-compR (BamHI)	AGTCGGATCCGTTGTAGACAGCCATGCGTA

Underlined italics: restriction sites.

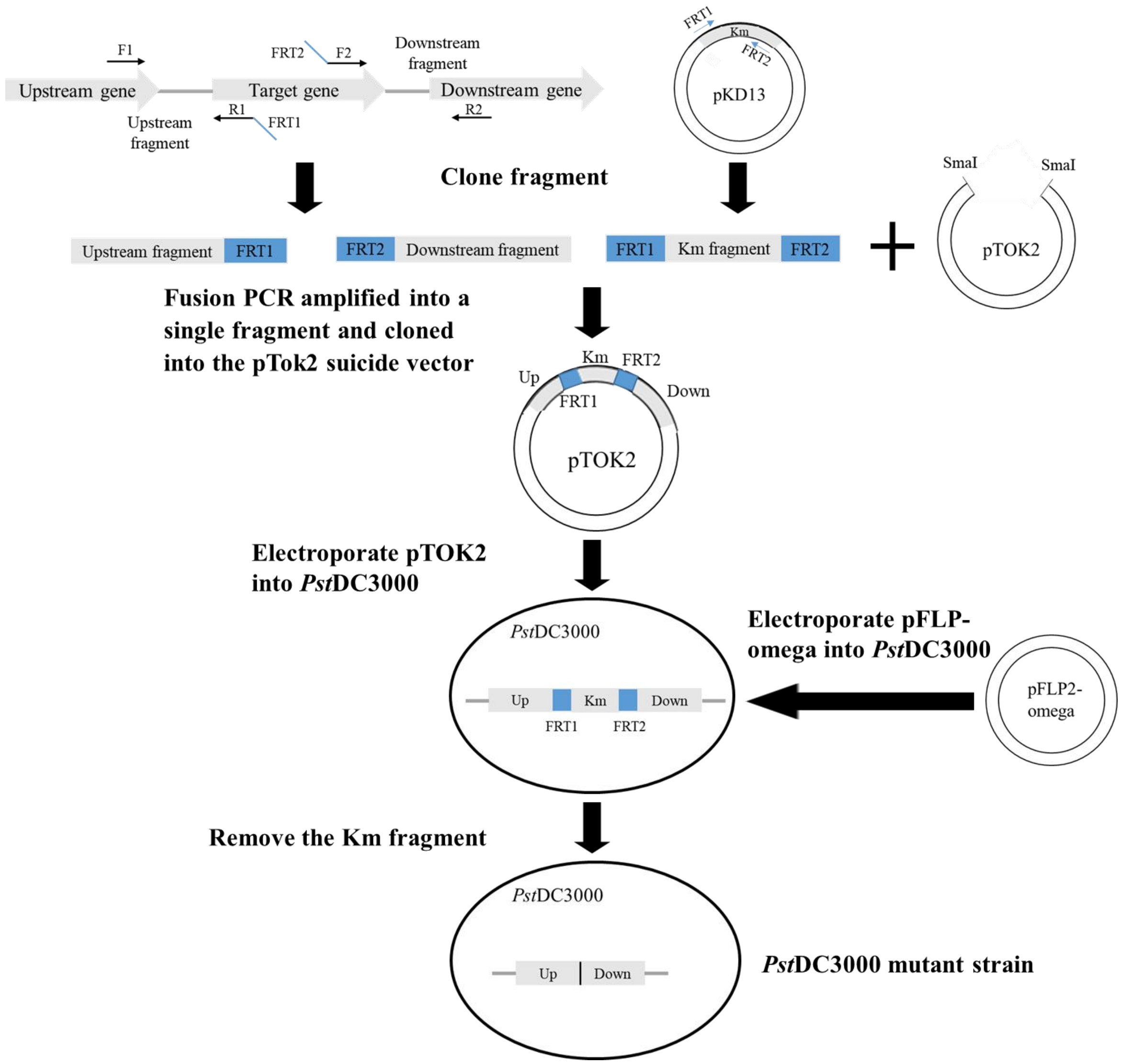

Figure S1. Schematic diagram for mutant construction by splicing overlap extension mutagenesis method.
